# Advancing Age Modulates Associations Between Cognitive Impairment and Brain Volumes in Early MS

**DOI:** 10.1002/acn3.70385

**Published:** 2026-04-10

**Authors:** Piriyankan Ananthavarathan, Marco Pitteri, Michael Foster, Sara Collorone, Sara Salama, Elisa Colato, Ferran Prados, Baris Kanber, Marios Yiannakas, Claudia A. M. Gandini Wheeler‐Kingshott, Indran Davagnanam, Frederik Barkhof, Declan Chard, Olga Ciccarelli, Ahmed Toosy

**Affiliations:** ^1^ Queen Square Institute of Neurology, Queen Square MS Centre, Department of Neuroinflammation University College London London UK; ^2^ National Hospital for Neurology and Neurosurgery, Department of Neuropsychology London UK; ^3^ Department of Neurology, Faculty of Medicine University of Alexandria Egypt; ^4^ Department of Medical Physics and Biomedical Engineering Faculty of Engineering Science, UCL Hawkes Institute, University College London London UK; ^5^ Universitat Oberta de Catalunya, e‐Health Centre Barcelona Spain; ^6^ The University of Pavia, Department of Brain and Behavioural Sciences Pavia Italy; ^7^ NIHR, University College London Biomedical Research Centre London UK; ^8^ Department of Radiology and Nuclear Medicine Amsterdam UMC Amsterdam the Netherlands; ^9^ UCL Queen Square Institute of Neurology, Department of Brain Repair and Rehabilitation London UK

**Keywords:** ageing, brain volume, cognitive impairment, grey matter volume, MACFIMS, MRI, multiple sclerosis

## Abstract

**Introduction:**

Cognitive impairment is common in multiple sclerosis (MS), but manifestations following the first demyelinating event are relatively unexplored. We investigated cross‐sectional associations between magnetic resonance imaging (MRI)–derived brain volumes and the presence of cognitive impairment outcomes five years after the first presentation with a demyelinating event.

**Methods:**

Comprehensive cognitive assessments were performed five years after a first clinical demyelinating presentation using the Minimal Assessment of Cognitive Function in MS (MACFIMS). Participant *Z* scores were calculated for each MACFIMS subtest using available regression‐based normative data. Participants were defined as having a cognitive outcome if one or more MACFIMS subtests were below the applied cutoff of *Z* ≤ −1.50. Gray matter volumes (GMV) were calculated using 3D T1‐weighted MRI sequences. Logistic regression models assessed associations between 5‐year MRI measures and cognitive impairment (adjusting for total intracranial volume where relevant).

**Results:**

Forty participants were recruited. Lower GMV at 5 years was associated with 5‐year cognitive impairment outcomes (OR = 1.06 per 1 cm^3^ decrease in GMV, 95% CI [1.00–1.13], *p* = 0.044). Among participants with a cognitive impairment outcome, there were significant associations between lower GMV and advancing age (*p* = 0.0095). Exploratory analysis of estimated marginal contrasts suggested that cognitive impairment outcomes were potentially associated with GMV above the age of 39 years (*Z* ≤ −1.50: *p* = 0.0476).

**Discussion:**

Five years after a first demyelinating event, GMV volumes on MRI are significantly associated with the presence of cognitive outcomes, while advancing age also appears to be associated with GMV and cognitive outcomes.

## 1 Introduction

Cognitive impairment is common in multiple sclerosis (MS), which can significantly affect quality of life [1, 2]. The estimated prevalence of MS‐related cognitive impairment varies between 34%–65% depending on disease course [3, 4, 5], with greater prevalence in advancing disease. Among those presenting with their first demyelinating event (i.e., clinically isolated syndrome, CIS), around 20%–25% are affected, rising to 30%–45% in relapsing‐remitting (RR) MS and 50%–75% in secondary progressive (SP) MS [3, 6]. Early diagnosis of MS‐related cognitive impairment is challenging, given its insidious onset, the diversity of symptoms, and the contribution of physical and psychosocial factors. Despite the heterogeneity of cognitive symptomatology, slower processing speed and memory deficits are more frequently observed [7, 8].

The pathobiological mechanisms responsible for cognitive dysfunction remain unclear, but contributions have been identified using magnetic resonance imaging (MRI). Although whole‐brain lesion loads from T2‐weighted MRI do not strongly correlate with cognitive impairment [9], there is evidence implicating the role of subcortical, juxtacortical, and gray matter lesions [10]. Furthermore, with additional MRI techniques, abnormalities in normal‐appearing white matter (e.g., magnetization transfer, diffusion tensor imaging and T1 relaxometry), cortical gray matter lesions (using double inversion recovery) [5, 11, 12, 13] and gray matter atrophy (both cortical and deep gray matter volume loss) [5, 14, 15] are also linked to cognitive outcomes.

Advancing age may also play a role in modulating pathobiological disease processes in MS and assist in explaining some of the phenotypic differences observed with disease progression [16]. Compared with younger individuals, those presenting later have less inflammatory (relapse) activity and are more likely to experience secondary progression [16] and neurodegeneration [17]. Yet, the relationship between advancing age and the presence of MS‐related cognitive outcomes remains poorly understood [18].

In this study, we investigated cross‐sectional relationships between brain MRI measures, age, and cognitive outcomes five years after the first clinical demyelinating event.

## 2 Materials and Methods

### 2.1 Participants

Clinical, cognitive, and MRI assessment data at 5 years following first presentation with a demyelinating event (i.e., a clinically isolated syndrome, CIS) were extracted from a prospective, longitudinal study. Participants were originally recruited within three months of their first clinical presentation with a demyelinating event from The National Hospital for Neurology and Neurosurgery, Queen Square or Moorfields Eye Hospital (London, UK). Eligible participants were aged between 18 and 65 years, able to undergo an MRI, undertake cognitive assessment in English, and had the capacity to provide written informed consent. Those with a history of other conditions affecting brain function (e.g., previous cerebrovascular accidents), psychoactive drug use, or those given an alternate diagnosis other than CIS or MS (e.g., MOGAD, NMOSD) were excluded. Ethical approval for the study protocol was obtained from the local Research Ethics Committee (13/LO/1762; 13/0231‐CIS2013).

### 2.2 Clinical Assessment

Clinical assessment included a thorough medical history, including details of clinical relapse and medication (including DMT), a neurological examination, and Expanded Disability Status Scale (EDSS) evaluation. Clinical assessment was deferred by at least 1 month in the event of a relapse occurring before a scheduled visit.

### 2.3 Cognitive Assessment

Cognitive outcomes were evaluated using the Minimal Assessment of Cognitive Function in MS (MACFIMS) [1]; a battery of tests that measure a comprehensive range of cognitive domains, including those mostly affected in MS (Table 1). For each participant, MACFIMS subtest raw scores were converted into *Z*‐scores using regression‐based normative data [19]. Following previously applied approaches [20, 21], impaired performance on each MACFIMS subtest was defined by a conventional threshold of *Z* ≤ −1.50. In line with previous exploratory work [22], participants were assigned as having a cognitive outcome (i.e., cognitive impairment) if ≥ 1 MACFIMS subtests fell below this *Z* score threshold in order to maximize the sensitivity of detecting cognitive impairments, which may otherwise go undetected (particularly given the paucity of prior cognitive research in CIS [23]). As cognitive function can be considered part of a spectrum, which can be affected more insidiously during the early stages of MS [23, 24], additional exploratory analyses were also undertaken in parallel using a lenient *Z* score cutoff (*Z* ≤ −1.00) to define subtler cognitive dysfunction outcomes and their correlation with MRI measures. Participants also underwent assessment for anxiety and depression using the Hospital and Anxiety Depression Scale (HADS) and fatigue levels using the Modified Fatigue Impact Scale (MFIS).

**TABLE 1:** Demographics, clinical and MRI characteristics at baseline and 5 years following first demyelinating event.

		Baseline (*n* = 40)	5 years (*n* = 40)
Age at baseline, years (mean, SD)		31.7 (7.39)	
Education at baseline, years (mean, SD)		16.4 ± 2.81	
Female sex, *n* (%)		26 (65%)	
CIS type, *n* (%)	Optic neuritis	34 (85%)	
	Brainstem/cerebellum	4 (10%)	
	Hemispheric	1 (2.5%)	
	Multifocal	1 (2.5%)	
Clinical Diagnosis, *n* (%)	CIS	25 (62.5%)	12 (30%)
	RRMS	15 (37.5%)	28 (70%)
EDSS, median (IQR)		1.50 (1–1.875)	1.25 (0–2)
DMT use by 5 years, *n* (%)	Total		22 (55%)
	Low efficacy		14 (35%)
	High efficacy		8 (20%)
Cognitive impairment diagnosis, *n* (%)	*Z* ≤ −1.50 (≥ 1 MACFIMS domain failed)		17 (43%)
	*Z* ≤ −1.00 (≥ 1 MACFIMS domain failed)		21 (53%)
MRI measures at 5 years	LC, median (IQR)		52.5 (28–99)
	LV, cm^3^ (mean, SD)		6.71 (6.72)
	GMV, cm^3^ (mean, SD)		664 (51.7)
	WMV, cm^3^ (mean, SD)		454 (45.0)
	TIV, cm^3^ (mean, SD)		1473 (118)

Abbreviations: CIS, clinically isolated syndrome; EDSS, expanded disability status scale; GMV, gray matter volume; IQR, interquartile range; LC, lesion count; LV, lesion volume; SD, standard deviation; TIV, total intracranial volume; WMV, white matter volume.

### 2.4 Image Acquisition

MRI sequences were acquired using a 3T Philips Ingenia CX scanner (Philips, Best, the Netherlands). The following sequences were acquired: isotropic three‐dimensional (3D) gradient‐echo T1‐weighted (3D‐T1w) [Acquisition time (AT) = 6:35 min, voxel size = 1 × 1 × 1 mm, flip angle (FA) = 8°, sagittal acquisition; TR = 7.0 ms, TE = 3.2 ms]; 3D Fluid‐Attenuated Inversion Recovery (3D‐FLAIR) [AT = 6:35 min, sagittal acquisition; voxel‐size = 1 × 1 × 1 mm, TR = 5000 ms, TE = 350 ms, TI = 1650 ms]; and 2D axial conventional spin‐echo proton density (PD)/T2‐weighted (T2w) images [AT = 3:41 min, voxel size = 1 × 1 × 3mm, TR = 3500 ms, TE = 19/85 ms].

Lesion masks were generated on 3D‐FLAIR sequences using an automated lesion segmentation pipeline (nicMS) [25, 26], manually quality‐checked and edited by M.A.F and S.S using a semi‐automated edge‐finding tool (3D Slicer v4.5.0–1 r24735 for Linux). Lesions smaller than 2 mm in any direction, or those found in areas typically associated with enlarged perivascular spaces (e.g., centrum semiovale), were excluded. 3D‐FLAIR and 3D T1w images were rigidly registered [27], following which lesion masks were then resampled into 3D T1w space. 3D T1w images were filled using a non‐local patch‐match lesion‐filling algorithm [27] and segmented into gray matter (including cortical and deep gray matter) and white matter using Geodesic Information Flows [28]. Binary segmentation masks were then used to calculate brain tissue volumes [gray matter (GMV), cortical gray matter (CGMV), deep gray matter (DGMV), and white matter (WMV) volumes], lesion counts (LC), lesion volumes (LV), and total intracranial volume (TIV). Analyses considering cortical gray matter (CGMV) and deep gray matter (DGMV) masks are presented in the supporting information.

### 2.5 Statistical Analysis

Statistical analysis was performed using IBM SPSS Statistics for Windows Version 28.0.0 (IBM, Armonk, NY) and R version 4.2.3 [29]. We sought to evaluate whether volumetric MRI measures (five years after a first demyelinating event) were associated with 5‐year cognitive outcomes, and whether age affected this relationship.

Binary logistic regression models were constructed in three stages, with 5‐year cognitive outcome (at either *Z* ≤ −1.50 or *Z* ≤ −1.00) as the dependent variable. Initially, simple models entered MRI measures alone as independent variables. Second, age and sex were added as additional covariates to determine their effects in combined models (as baseline education was already adjusted for in MACFIMS regression‐based norms, this was not included again as an additional covariate). Interaction effects between age, cognitive outcomes, and MRI measures were also investigated in separate models. Linear models (without interaction) were assessed for multicollinearity by examining the variance inflation factors (VIF), (with a VIF > 5 considered abnormal).

Additional, post hoc, logistic regression analyses were also built to determine whether other clinical features (e.g., DMT or steroid use, clinical relapse frequency, fatigue, or anxiety/depression) were associated with binary 5‐year cognitive outcomes. Post hoc linear regression models explored whether DMT use was significantly associated with 5‐year MRI measures.

Two‐sided hypothesis testing was used at significance level of ≤ 0.05 for statistical inferences. All models considering volumetric MRI measures (i.e., GMV, WMV, LV) were adjusted for TIV [30]. As lesion volumes increase and volumetric brain measures (GMV, WMV) decrease with advancing disease, we present the inverse function of volumetric brain measures in linear regression models to reflect the increasing likelihood of developing a cognitive outcome per cm^3^ decrease in GMV and WMV. Conversely, for lesion volume, results are presented as the likelihood of developing a cognitive outcome per cm^3^ increase.

## 3 Results

### 3.1 Demographics, Clinical, and MRI Characteristics

Clinical, MRI, and cognitive data were available for 40 participants (26 females, 65%). The mean age at the time of the 5‐year assessment was 36.5 ± 6.72 years. At the time of the first demyelinating event, 15 (37.5%) participants were diagnosed with MS (using the revised 2017 McDonald criteria [31]); by 5 years after the first demyelinating event, this increased to 28 (70%) (Table 1). Median EDSS was 1.50 at baseline and 1.25 at 5 years. At 5 years, with conventional thresholds (*Z* ≤ −1.50), 17 (43%) participants were classified as cognitively impaired (failing ≥ 1 MACFIMS domain), whilst at the more lenient (*Z* ≤ −1.00) threshold, 21 (53%) participants were classified as cognitively impaired See Supplementary Tables S5 and S6.

Twenty‐two (55%) participants received at least one DMT by 5 years, eight of whom (22%) received a high‐efficacy agent. Sixteen participants (40%) received at least one course of steroids following the first demyelinating event or at a subsequent relapse. Using post hoc logistic regression, cognitive outcomes (at either *Z* score threshold) were not associated with DMT use or type (high‐ versus low‐efficacy), steroid use, relapse frequency, fatigue (total Modified Fatigue Impact Scale), or anxiety/depression (Hospital Anxiety and Depression Scale). DMT use or type was also not associated with 5‐year MRI measures in post hoc linear regression models.

Cognitive performance was most frequently below cutoff on the Controlled Oral Word Association Test (COWAT), a test of phonemic fluency sensitive to executive functioning [32] (*Z* ≤ −1.50: 7 participants failed, *Z* ≤ −1.00, 11 participants failed), and the Delis–Kaplan Executive Function System (DKEFS) Sorting Descriptive Score, a further measure of higher‐order executive functioning (*Z* ≤ −1.50: 11 participants failed, *Z* ≤ −1.00, 14 participants failed) (Table 2).

**TABLE 2:** Cognitive domains tested by each MACFIMS component and spread of participants failing tests.

MACFIMS subtests	Cognitive domains tested	Number of participants failing subtest at 5‐year assessment (%)	
		*Z* ≤ −1.00	*Z* ≤ −1.50
Controlled oral word association test (COWAT)	Verbal fluency, executive functioning	11 (28%)	7 (18%)
California verbal learning test (CVLT‐II) – Total learning (Immediate recall trials 1–5)	Verbal learning	0 (0%)	0 (0%)
California verbal learning test (CVLT‐II) delayed recall	Verbal recall memory	0 (0%)	0 (0%)
Brief visuospatial memory test‐revised (bvmtr) – total learning (Immediate recall trials 1–3)	Visual learning	0 (0%)	0 (0%)
Brief visuospatial memory test‐revised (bvmtr) – delayed recall	Visual recall memory	1 (3%)	0 (0%)
Paced auditory serial addition test (PASAT) −3 s	Sustained attention, auditory working memory, information processing speed, calculation (multi‐domain)	2 (5%)	1 (3%)
Paced auditory serial addition test (PASAT) −2 s		0 (0%)	0 (0%)
Symbol digit modalities test (SDMT)	Attention, processing speed	1 (3%)	0 (0%)
Delis–Kaplan executive function system (dkefs‐sorting): correct scores	Executive functioning	2 (5%)	0 (0%)
Delis–Kaplan executive function system (dkefs‐sorting): description score	Executive functioning	14 (35%)	11 (28%)
Benton judgement of line orientation (jlo)	Visuospatial perception	2 (5%)	1 (3%)

### 3.2 Cross‐Sectional Associations Between 5‐Year MRI Measures and 5‐Year Cognitive Outcome

At *Z* ≤ −1.50, logistic regression models demonstrated that a decrease in GMV or CGMV was associated with increasing odds of developing a cognitive outcome at 5 years (see Table 3, and Table S7): per 1cm^3^ decrease in GMV, OR = 1.06, 95% CI [1.00–1.13], *p* = 0.044. The mean GMV among the cognitively impaired group (*Z* ≤ −1.50) was 659.23 ± 56.28 cm^3^, while in the cognitively preserved group the mean volume was 668.58 ± 49.01 cm^3^. Cognitive outcomes (*Z* ≤ −1.50) were not associated with other 5‐year MRI measures (DGMV, WMV, LC or LV). In combined models that which considered associations between 5‐year measures, age, and sex with cognitive outcomes, all covariates lost significance.

**TABLE 3:** Logistic regression models with MRI measures predicting 5‐year **cognitive outcome (adjusted for TIV)**.

Odds ratio [95% CI] of developing cognitive outcome per cm^3^ decrease in GMV/WMV volumetric MRI measures; per cm^3^ increase in lesion volume; or per increase in lesion count							
*Z* score cutoff for cognitive outcome	MRI measure	Independent models (5‐year cognitive outcome, adjusted for TIV alone)			Combined models (5‐Year cognitive outcome, adjusted for TIV, age, sex)		
		Odds ratio [95% CI]	*p*	*R* ^2^	Odds ratio [95% CI]	*p*	*R* ^2^
*Z* ≤ −1.50	GMV	1.06 [1.00–1.13]	0.044*	0.152	1.05 [0.980–1.12]	0.169	0.288
	WMV	0.99 [0.951–1.03]	0.650	0.007	0.985 [0.941–1.03]	0.519	0.245
	LV	1.06 [0.952–1.18]	0.287	0.042	1.00 [1.00–1.00]	0.501	0.246
	LC	0.99 [0.975–1.00]	0.141	0.075	0.988 [0.973–1.01]	0.166	0.286
*Z* ≤ −1.00	GMV	1.06 [1.00–1.13]	0.041*	0.177	1.05 [0.982–1.11]	0.162	0.282
	WMV	1.00 [0.960–1.04]	0.987	0.025	0.995 [0.952–1.04]	0.815	0.227
	LV	1.04 [0.941–1.14]	0.465	0.043	1.00 [1.00–1.00]	0.750	0.228
	LC	0.99 [0.972–1.00]	0.095	0.099	0.985 [0.968–1.00]	0.100	0.295

Abbreviations: CI, confidence interval; GMV, gray matter volume; LC, lesion count; LV, lesion volume; OR, odds ratio; TIV, total intracranial volume; WMV, white matter volume.

* *p* < 0.05.

When modeled independently, age was significantly associated with 5‐year cognitive outcomes (*Z* ≤ −1.50): OR = 1.15 per year of advancing age, 95% CI [1.02–1.29], *p* = 0.022; as well as with 5‐year GMV (β −0.636 cm^3^ per year of advancing age, 95% CI [−1.17, −1.03], *p* = 0.021).

Using the more lenient threshold (Z ≤ −1.00) for cognitive outcomes, logistic regression models again demonstrated that decreases in 5‐year GMV were significantly associated with an increasing likelihood of a cognitive outcome (Table 3): per 1cm^3^ decrease in GMV OR = 1.06, 95% CI [1.00–1.13], *p* = 0.041. The mean GMV among the cognitively impaired group (*Z* ≤ −1.00) was 667.01 ± 53.85 cm^3^, while in the cognitively preserved group, the mean GMV was 661.94 ± 50.62 cm^3^. Cognitive outcomes (*Z* ≤ −1.00) were not associated with other 5‐year MRI measures (DGMV, WMV, LV, and LC), except CGMV (see Table S7), while in combined models, MRI measures, age and sex were no longer significantly associated with 5‐year cognitive outcomes.

Linear regression models considering the inverse (i.e., 5‐year binary cognitive outcomes associated with 5‐year MRI measures) are presented in the supporting information (see Table S8). All significant linear regression models demonstrated a VIF < 1.5 (see Table S9).

### 3.3 Interaction Effects of Age and Cognitive Outcomes With GMV at 5 Years

At both *Z* score thresholds, there were borderline significant interaction effects between age and cognitive outcomes (*Z* ≤ 1.50 slope interaction *p* = 0.046; Z ≤ 1.00 slope interaction *p* = 0.054). Among individuals defined as cognitively impaired, greater age was associated with significantly lower GMV at either cutoff (*Z* ≤ −1.50: β change in GMV −0.946 cm^3^ per year of advancing age, *p* = 0.0095; *Z* ≤ −1.00: β change in GMV −0.908 cm^3^ per year of advancing age, *p* = 0.01). In contrast, no significant associations were observed between age and GMV among those who did not develop a cognitive outcome (*Z* ≤ −1.50: *p* = 0.509; *Z* ≤ −1.00: *p* = 0.529). Figure 1 demonstrates the significant negative slope in GMV with advancing age among those with a cognitive outcome at 5 years (*Z* ≤ −1.50) (highlighted in blue), in contrast to those without a cognitive outcome (highlighted in red). For *Z* ≤ −1.00 figure, please refer to Figures S3 and S4.

**FIGURE 1:**
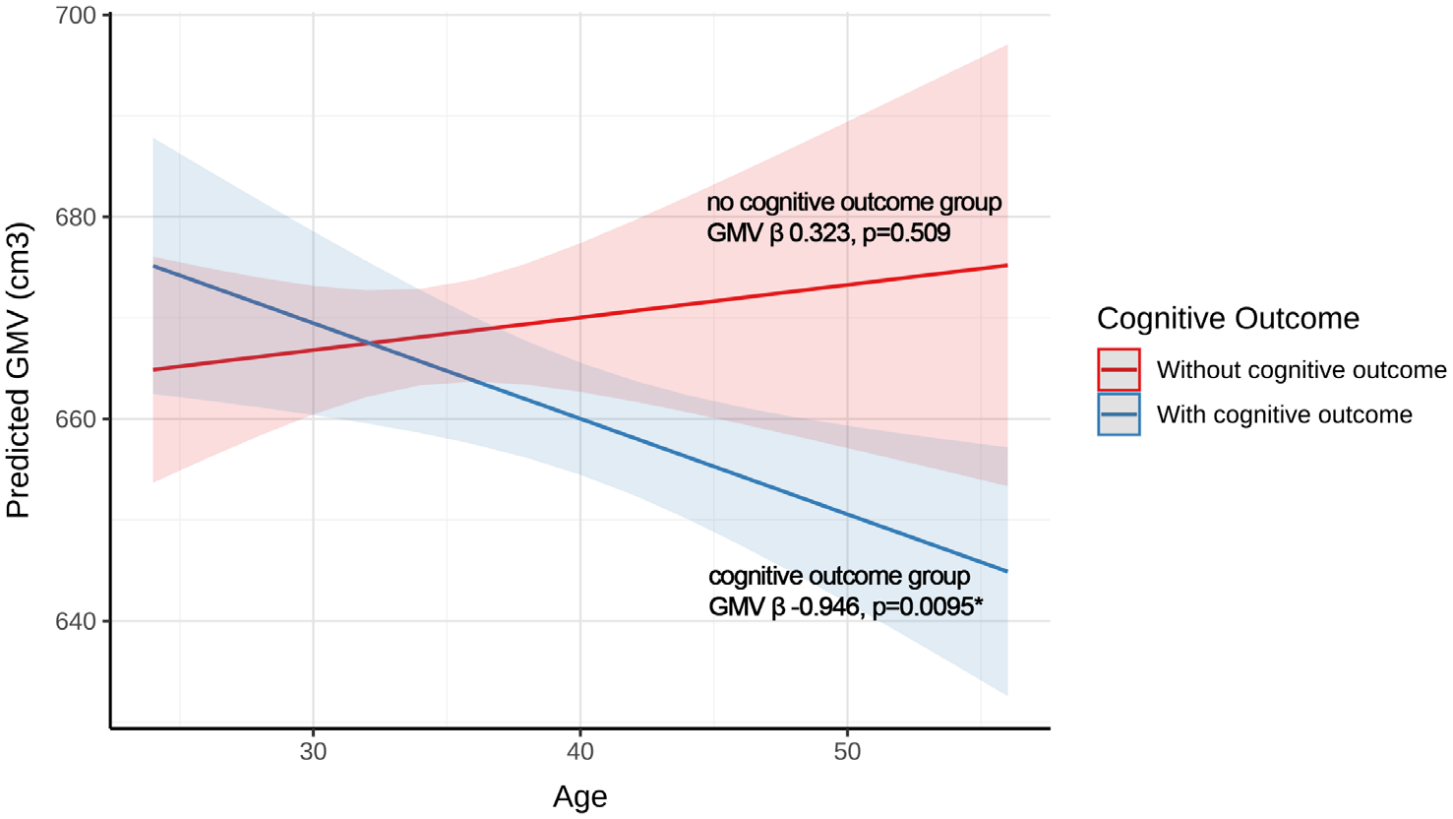
Adjusted GMV 5 years after CIS onset versus age with interaction effects of cognitive impairment outcomes (*Z* ≤ −1.50).

Additional, exploratory, post hoc marginal contrast analyses suggested that, at either Z score cutoff, there were potentially significant associations between cognitive outcomes and GMV above the age ≥ 39 years (at age 39 years: *Z* ≤ −1.50: *p* = 0.0476, and *Z* ≤ −1.00: *p* = 0.0364) (Table 4).

**TABLE 4:** Interaction effects of age and cognitive outcomes (at either Z‐score cutoff) towards GMV.

*Z* score cutoff	Age	β change [95% CI] in GMV (*cm* ^*3*^) (adjusted for TIV) *with development of cognitive outcome at either Z score*	*p*
*Z* ≤ −1.50	25	−9.01 [−25.3, 7.28]	0.269
	35	3.68 [−4.51, 11.9]	0.368
	38	7.49 [−0.776, 15.8]	0.074
	39	8.76 [0.0994, 17.41]	0.048*
	40	10.02 [0.821, 19.2]	0.034*
	45	16.37 [2.94, 29.8]	0.018*
*Z* ≤ −1.00	25	−7.77 [−23.65, 8.1]	0.327
	35	4.42 [−3.58, 12.4]	0.270
	38	8.08 [−0.157, 16.3]	0.054
	39	9.30 [0.623, 18.0]	0.036*
	40	10.5 [1.26, 19.8]	0.027*
	45	16.6 [3.03, 30.2]	0.018*

Abbreviations: CI, confidence interval; GMV, gray matter volume; TIV, total intracranial volume.

* *p* < 0.05.

Among participants who developed a cognitive impairment at 5 years for either *Z* score cutoff, there were significant differences in 5‐year GMV between age 25 and 45 (*Z* ≤ −1.50: ΔGMV 16.4 cm^3^, *p* = 0.0184; *Z* ≤ −1.00: ΔGMV 16.6 cm^3^, *p* = 0.018). Conversely, among participants who remained cognitively preserved, there were no associations between 5‐year GMV and age (Z ≤ −1.50: ΔGMV −9.01 cm^3^, *p* = 0.269; *Z* ≤ −1.00: ΔGMV −7.77 cm^3^, *p* = 0.327). Figure 2 depicts the significant modeled differences in GMV at age 25 and 45 among those with a cognitive outcome (*Z* ≤ −1.50), in comparison to the lack of any difference in GMV observed between age groups among those who did not develop a cognitive outcome. For *Z* ≤ −1.00 figure, please refer to Figures S3 and S4.

**FIGURE 2:**
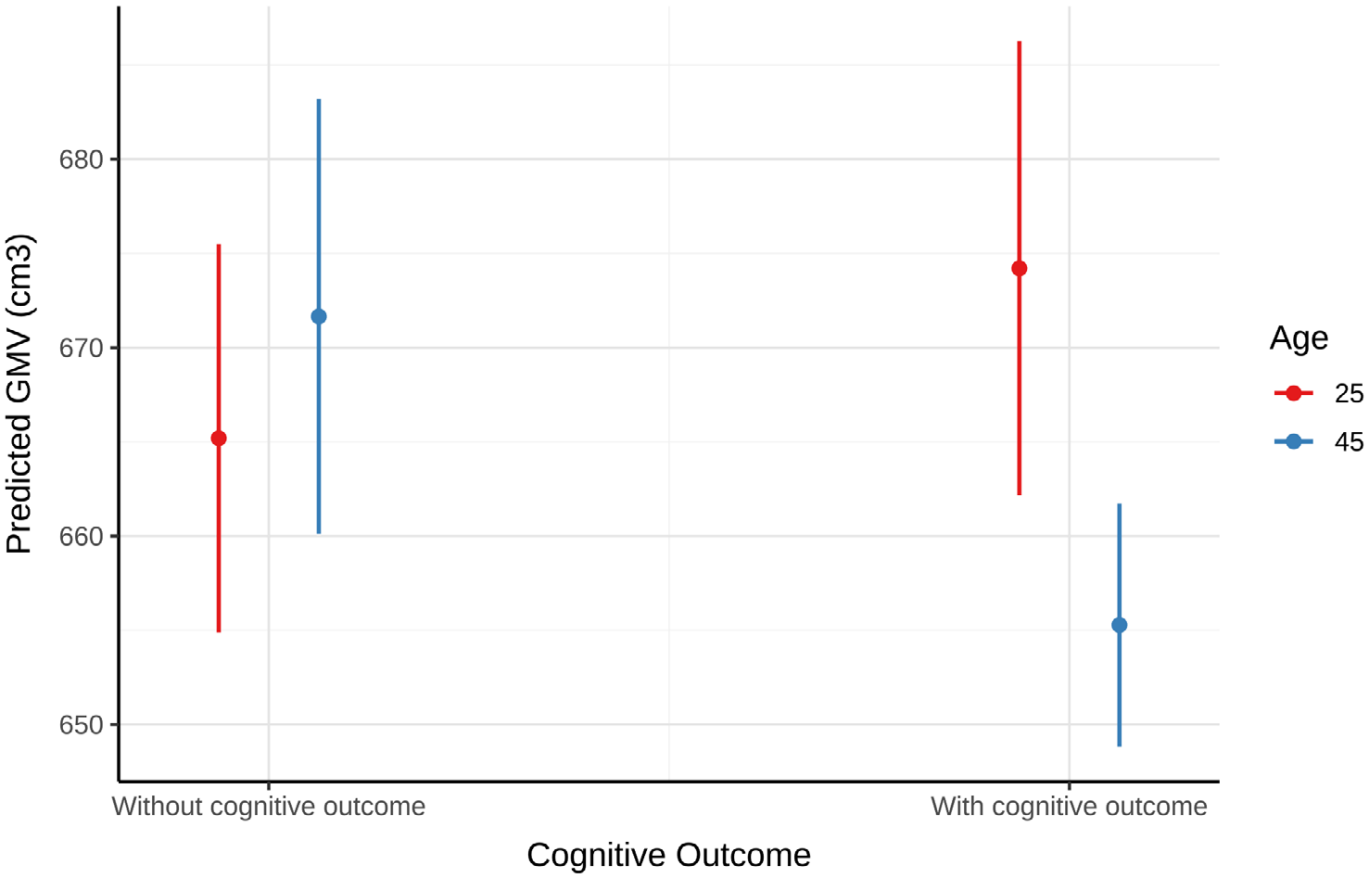
Adjusted GMV 5 years after CIS onset versus cognitive impairment outcomes (*Z* ≤ −1.50) with interaction effects of age.

## 4 Discussion

We investigated cross‐sectional relationships between brain MRI measures, age and cognitive impairment. Our results showed that, five years after a first clinical demyelinating event, GMV (and CGMV) MRI measures are significantly associated with the presence of cognitive impairment using either a conventional definition of impairment (failure of ≥ 1 MACFIMS cognitive subtest with *Z* ≤ −1.50) or a more lenient one (failure of ≥ 1 MACFIMS cognitive subtest with *Z* ≤ −1.00) and corroborate existing literature [14, 15]. Among individuals who showed reduced cognitive outcomes at 5 years, advancing age also appeared to be associated with lower GMV (and CGMV), even though cognitive *Z* scores were already adjusted for age and sex. We speculate that this observation could relate to additional effects of brain aging on gray matter brain volumes among individuals who developed cognitive impairment after their first demyelinating event [33, 34], although further longitudinal analyses and/or brain‐age modeling should investigate this in the future. In contrast, we found that individuals with intact cognitive function at 5 years demonstrated a relative preservation of gray matter volumes independent of age. Furthermore, at ages below 39 years, there were no discernible differences in gray matter brain volumes between participants who were either cognitively impaired or cognitively preserved. Our results suggest age potentially interacts with the associations between MS‐related cognitive outcomes and brain volumes. These may suggest a potentially synergistic effect between brain aging and lower gray matter volumes on the development of reduced cognitive outcomes, and wonder whether a decrease in gray matter reserve may contribute to modulating MS‐related cognitive outcomes with advancing age. This is relevant, given the increasing awareness of accelerated (or premature) brain ageing in MS (brain‐age paradigm) [35, 36]. Given that disease and cognitive progression can occur in the absence of clinically or radiologically evident relapses [37], future pre‐symptomatic longitudinal studies may help to understand early pathobiological mechanisms and clinical disease evolution along the MS timeline even before the first demyelinating event (e.g., radiologically isolated syndromes).

Our results also demonstrate that executive dysfunction was the most frequent cognitive deficit among our study cohort, whereas SDMT abnormalities were less prominent. This may be due to the fact that larger MS cohorts may perhaps underestimate executive dysfunction consequent to the use of brief screening measures (e.g., BICAMS) or batteries that lack dedicated executive measures (e.g., the Brief Repeatable Battery of Neuropsychological Tests, BRB‐NT). In contrast, our use of the MACFIMS battery arguably enhanced sensitivity to executive deficits. Specifically, the D‐KEFS Sorting Test is widely recognized as a sensitive measure of higher‐order executive function, requiring abstraction, concept formation, and cognitive flexibility. Behavioral studies suggest that the two main components of D‐KEFS differ in sensitivity: the Description Score reflects higher‐order functions, i.e., metacognitive insight and verbal abstraction, whereas Correct Sorts may partly reflect trial‐and‐error strategies. Fine and colleagues [38] found that Description Scores were more strongly associated with executive impairment than Correct Sorts, highlighting their utility in detecting subtle deficits. These findings align with Parmenter and colleagues [39], who supported the D‐KEFS Sorting Test's sensitivity to executive dysfunction in MS, demonstrating its ability to distinguish patients from controls beyond processing speed or mood effects. In another study, Migliore et al. [40] showed that the Description Score was more sensitive than Correct Sorts in identifying executive deficits in mildly disabled RRMS patients, suggesting its value in early disease stages. Additionally, Pitteri et al. [32] demonstrated that phonemic fluency reveals executive dysfunction even when SDMT performance is intact, underlining that executive deficits may emerge independently of processing speed impairments.

Although the application of conventional cutoffs, such as the 5th percentile or *Z* ≤ −1.50 [5, 19, 20, 21], is helpful in defining cognitive outcomes, an alternative approach can derive from treating cognitive dysfunction as a continuum that ranges from normal cognition to severe cognitive impairment. Such thresholds used to determine abnormal cognitive performance do not necessarily reflect what is either functionally significant to a person with MS nor distinguish clearly separate groups (e.g., as would be the case for a bimodal distribution). Thus, individuals with MS who may not necessarily reach conventional definitions of cognitive impairment may still experience functional difficulties with activities of daily living which may reduce their quality of life. This argument informed our investigation into applying both conventional and more lenient thresholds (i.e., *Z* ≤ −1.50 and *Z* ≤ −1.00) to assess a broader proportion of MS individuals performing lower than expected in cognitive tests. The utility of this approach was highlighted by the fact that models in which baseline GMV was associated with 5‐year cognitive outcomes only became significant by adopting the more sensitive cutoff (i.e., *Z* ≤ −1.00), compared with the more conventional one (*Z* ≤ −1.50).

Our study had several limitations. The relatively small sample size may explain why associations between 5‐year MRI measures and 5‐year cognitive outcomes were only identified in gray matter volumes (GMV and CGMV), while no associations were identified with other MRI measures (e.g., DGMV). This may be due to the study potentially being underpowered and the absence of association should not be interpreted as evidence of absence. Also, while we explored the effects of general volumetric brain measures (e.g., GMV, WMV), other specific brain structures (e.g., hippocampus, thalamus, brainstem/cerebellum) may also play significant roles in the accrual of cognitive impairment. Future regional analyses considering these structures in more detail would therefore be warranted to better understand their relationships with cognitive outcomes. Additionally, while our results demonstrated significant differences in GMV, it is unclear whether the magnitude of the observed effect is biologically meaningful and therefore should also be interpreted with caution. Furthermore, the lack of healthy control data (e.g., MRI measures, cognitive data), or more detailed information regarding DMT or steroid use may also limit interpretation of study results. However, these limitations do not necessarily weaken the significant associations already observed in the study, nor do they materially alter the interpretation of findings. Second, a high proportion of our study cohort presented with optic neuritis as their initial demyelinating event. Hence, it is important to exercise caution when interpreting our results with respect to a more typical CIS population. As the study only explored early disease evolution and the onset of 5‐year cognitive impairment (using the MACFIMS), it is of uncertain clinical relevance how many participants identified as developing cognitive dysfunction using the lenient cutoff (*Z* ≤ −1.00) would subsequently have developed more evident cognitive impairment later in the disease course. Hence, longer‐term follow‐up data and further longitudinal studies are warranted to determine whether exploratory cutoffs (e.g., *Z* ≤ −1.00) may be associated with subsequent onset of clinically‐evident cognitive impairment using conventional definitions (i.e., *Z* ≤ −1.50) over time [41]. It is also important to mention that cognitive impairment classifications using tools such as the MACFIMS remain arbitrary, with no unified consensus to determine what constitutes a cognitive impairment. As such, in the present study, we defined cognitive impairment as failure of at least one MACFIMS subtest at either *Z* score cutoff. Applying a more negative *Z* score cutoff with a greater number of failed MACFIMS subtests can increase specificity but lower sensitivity, and this could have significantly underpowered the present study (for instance, only two participants would have been defined as cognitively impaired, based on failure of ≥ 2 MACFIMS cognitive subtests at *Z* ≤ −1.50; see Supporting Information). Also, testing cognitive function using validated tools [8] such as the MACFIMS are still not routinely performed in clinical practice, despite cognitive symptoms being a frequent complaint among patients with CIS or MS [1, 2], and hence it may be argued that cognitive assessments should be incorporated into routine early diagnostics and clinical surveillance, with particular attention to higher and lower order executive functions. We also note the low rate of SDMT subtest failure among the study cohort (at *Z* ≤ −1.00 3% had failed, at *Z* ≤ −1.50 0% had failed), which could be explained by a number of reasons such as ceiling effects, particularly among those with a higher level of cognitive reserve (and hence, relative preservation in SDMT compared with population norms), practice effects (wherein the original prospective study was based on relatively short retesting intervals at 0, 1, 3 and 5 year timepoints), as well as due to heterogeneity in the domains affected within the relatively small study sample.

To conclude, our study demonstrates that five years after a first demyelinating event, GMV (and CGMV) MRI measures were lower among individuals who developed cognitive impairment compared with those who did not. Uniquely, our results also suggest advancing age and cognitive outcomes are potentially related in their association with gray matter brain volumes.

## Author Contributions

P.A. project conception, data collection and analysis, Statistical analysis, preparation and revision of manuscript. M.P. project conception, preparation and revision of manuscript. M.F. data collection, MRI/Image analysis, manuscript review. S.C. data collection, manuscript review. S.S. data collection, manuscript review. E.C. data collection, manuscript review. F.P. MRI/Image analysis, manuscript review. B.K. MRI/Image analysis, manuscript review. M.Y., MRI/Image analysis, manuscript review. C.A.M.G.W.‐K. MRI/Image analysis, manuscript review. I.D. MRI/Image analysis, manuscript review. F.B. MRI/Image analysis, manuscript review. D.C. statistical analysis, preparation and revision of manuscript. O.C. data collection and analysis, MRI/Image analysis, preparation and revision of manuscript. A.T. project conception, data collection and analysis, statistical analysis, MRI/Image analysis, preparation and revision of manuscript.

## Funding

This study was supported by the Medical Research Council (MRC) UK (Grant Reference: MR/S026088/1), NIHR BRC (541/CAP/OC/818837), and RoseTrees Trust (A1332 and PGL21/10079).

## Ethics Statement

Ethical approval was obtained from the Local Research Ethics Committee who approved the study protocol (13/LO/1762; 13/0231‐CIS2013).

## Conflicts of Interest

PA—Clinical research fellow funded by an MS Society grant (Ref: 141). Previously in a post supported by Merck (supervised by D. Chard and SA Trip).

MP—No disclosures.

MF—supported by a grant from the MRC (MR/S026088/1) and has received speaker honoraria from Merck.

SC—supported by the RoseTrees Trust (A1332 and PGL21/10079) and has received speaker honoraria from Merck.

SS—supported by MSIF McDonald Fellowship/ARSEP.

EC—No disclosures.

FP—receives funding from the National Institute for Health Research (NIHR), Biomedical Research Centre initiative at University College London Hospitals (UCLH), and previously received a Guarantors of Brain fellowship 2017–2020.

BK– Receives partial funding from BRC NIHR Funding Initiative at UCL and UCLH.

MY—No disclosures.

CWK—receives funding from the MS Society (#77), Wings for Life (#169111), BRC (#BRC704/CAP/CGW), UCL Global Challenges Research Fund (GCRF), MRC (#MR/S026088/1) and Ataxia UK, and is a shareholder in Queen Square Analytics Ltd.

ID—No disclosures.

FB—supported by the NIHR biomedical research centre at UCLH. Steering committee or Data Safety Monitoring Board member for Biogen, Merck, ATRI/ACTC and Prothena. Consultant for Roche, Celltrion, Rewind Therapeutics, Merck, IXICO, Jansen, Combinostics. Research agreements with Merck, Biogen, GE Healthcare, Roche. Co‐founder and shareholder of Queen Square Analytics Ltd.

DC—consultant for Hoffmann‐La Roche. In the last three years he has been a consultant for Biogen, has received research funding from Hoffmann‐La Roche, the International Progressive MS Alliance, the MS Society, the Medical Research Council, and the National Institute for Health Research (NIHR), University College London Hospitals (UCLH) Biomedical Research Centre, and a speaker's honorarium from Novartis. He co‐supervises a clinical fellowship at the National Hospital for Neurology and Neurosurgery, London, which is supported by Merck.

OC—serves on a DSMB for Novartis, has acted as a consultant for Biogen, and has received speaker honoraria from Merck. She is funded by the NIHR Research Professorship (RP‐2017‐08‐ST2‐004).

AT—speaker honoraria from Merck, Biomedia, Sereno Symposia International Foundation, Bayer and At the Limits and meeting expenses from Merck, Biogen Idec and Novartis. He has been supported by recent grants from the Medical Research Council UK (MR/S026088/1), NIHR BRC (541/CAP/OC/818837) and RoseTrees Trust (A1332 and PGL21/10079). Associate editor for Frontiers in Neurology (Neuro‐ophthalmology section) and on the editorial board for Neurology and Multiple Sclerosis Journal.

## Supporting information


**Table S5:** Logistic regression models of fatigue, anxiety and depression scores independently predicting 5‐year cognitive outcomes.
**Table S6:** Number of participants defined as having cognitive impairment based on 1 vs. 2 domains.
**Table S7:** Additional logistic regression models with CGMV and DGMV MRI measures predicting 5‐year cognitive outcome.
**Table S8:** Linear regression models with cognitive outcome predicting 5‐year MRI measures.
**Table S9:** Variance Inflation Factors for significant linear regression models (GMV, CGMV).
**Figure S3:** Adjusted GMV 5 years after CIS onset versus Age with Interaction effects of cognitive Impairnebt Outcomes (Z < −1.00).
**Figure S4:** Adjusted GMV 5 years after CIS onset versus Cognitive Impairmnet Outcomes (Z < −1.00) with Interaction Effects of age.

## Data Availability

The data that support the findings of this study are available from the corresponding author upon reasonable request.
