# Supplementary material for: Advancing Age Modulates Associations Between Cognitive Impairment and Brain Volumes in Early MS
Source: Ann Clin Transl Neurol. 2026 Apr 10;13(10):2124–33. doi: 10.1002/acn3.70385 (PMC13394131; doi:10.1002/acn3.70385)
Supplement: Supplementary file 1 [file ACN3-13-2124-s001.docx]

**Supplementary Materials**

| **SUPPLEMENTARY TABLE 5**  **Logistic regression models of fatigue, anxiety and depression scores independently predicting 5-year cognitive outcomes** | | | |
| --- | --- | --- | --- |
| **Z-score cutoff** | **Predictor** | **OR [95% CI]  per unit increase on assessment score** | **p=** |
| **Z≤-1.50** | **MFIS (Total Score)** | 1.01 [0.975-1.04] | 0.617 |
|  | **HADS-A** | 0.975 [0.846-1.12] | 0.726 |
|  | **HADS-D** | 1.07 [0.925-1.24] | 0.369 |
| **Z≤-1.00** | **MFIS (Total Score)** | 0.998 [0.965-1.03] | 0.898 |
|  | **HADS-A** | 1.00 [0.869-1.15] | 0.996 |
|  | **HADS-D** | 1.04 [0.897-1.20] | 0.630 |
| Legend: HADS = Hospital Anxiety and Depression Score, MFIS = Modified Fatigue Impact Scale | | | |

| **SUPPLEMENTARY TABLE 6**  **Number of participants defined as having cognitive impairment based on 1 vs. 2 domains** | | |
| --- | --- | --- |
| **Z-score cutoff** | **Number (%) of participants failing  ≥1 MACFIMS subtests** | **Number (%) of participants failing  ≥2 MACFIMS subtests** |
| **Z≤-1.50** | 17 (43%) | 2 (5%) |
| **Z≤-1.00** | 21 (53%) | 7 (18%) |

| **SUPPELEMENTARY TABLE 7**  **Additional logistic regression models with CGMV and DGMV MRI measures predicting 5-year cognitive outcome** | | | | | | | |
| --- | --- | --- | --- | --- | --- | --- | --- |
|  | | **Odds Ratio [95% CI] of developing cognitive outcome**  *per cm^3^ decrease in CGMV/DGMV volumetric MRI measures* | | | | | |
| **Z-score cutoff for cognitive outcome** | **MRI measure** | **INDEPENDENT MODELS (5-year cognitive outcome,  adjusted for TIV alone)** | | | **COMBINED MODELS**  **(5-Year cognitive outcome,  adjusted for TIV, Age, Sex)** | | |
|  |  | **Odds Ratio [95% CI]** | **p=** | **R^2^** | **Odds Ratio [95% CI]** | **p=** | **R^2^** |
| **Z ≤ -1.50** | CGMV | **1.07 [1.01-1.14]** | **0.033*** | 0.170 | 1.05 [0.985-1.12] | 0.126 | 0.302 |
|  | DGMV | 0.81 [0.529-1.25] | 0.343 | 0.031 | 0.75 [0.469-1.19] | 0.223 | 0.275 |
| **Z ≤ -1.00** | CGMV | **1.07 [1.00-1.13]** | **0.035*** | 0.185 | 1.05 [0.984-1.12] | 0.137 | 0.290 |
|  | DGMV | 0.91 [0.596-1.39] | 0.655 | 0.031 | 0.847 [0.541-1.33] | 0.470 | 0.240 |
| Legend: CI = Confidence Interval, CGMV = Cortical Grey Matter Volume, DGMV = Deep Grey Matter Volume, OR = Odds Ratio,  TIV = Total Intracranial Volume | | | | | | | |

| **SUPPLEMENTARY TABLE 8**  **Linear regression models with cognitive outcome predicting 5-year MRI measures** | | | | | |
| --- | --- | --- | --- | --- | --- |
|  | | **β change [95% CI] in MRI measure with development of CI** *[GMV, CGMV, DGMV, WMV, LV: cm^3^ change in MRI measure, LC: change in number of lesions]* | | | |
| **Z-score cutoff** | **5-year MRI measure predicted** | **INDEPENDENT MODELS (Predictor: 5-year Cognitive Outcome, TIV)** | **p=** | **COMBINED MODELS**  **(Predictors: 5-Year Cognitive Outcome, TIV, Age, Sex)** | **p=** |
| **Z≤-1.50** | GMV | **-8.44 [-16.3, -0.605]** | **0.035*** | 5.53 [-14.0, 2.88] | 0.191 |
|  | CGMV | **-8.89 [-16.6, -1.16]** | **0.025*** | -6.20 [-14.56, 2.16] | 0.141 |
|  | DGMV | 0.458 [-0.536, -1.45] | 0.357 | 0.663 [-0.447, 1.77] | 0.233 |
|  | WMV | 2.26 [-8.17, 12.7] | 0.663 | 2.90 [-8.83, 14.6] | 0.619 |
|  | LC | 21.7 [-7.28, 50.7] | 0.138 | 24.1 [-8.60, 56.7] | 0.144 |
|  | LV | -2.32 [-6.72, 2.09] | 0.293 | -1.42 [-6.33, 3.50] | 0.562 |
| **Z≤-1.00** | GMV | **-8.57, [-16.4, -0.760]** | **0.032*** | -5.57 [-13.9, 2.74] | 0.183 |
|  | CGMV | **-8.78, [-16.5, -1.04]** | **0.027*** | -5.93 [-14.2, 2.34] | 0.154 |
|  | DGMV | 0.215 [-0.788, 1.22] | 0.666 | 0.369 [-0.743, 1.48] | 0.505 |
|  | WMV | 0.073 [-10.4, 10.5] | 0.989 | 0.613 [-11.0, 12.2] | 0.915 |
|  | LC | 25.9 [-3.06, 54.8] | 0.495 | 28.7 [-3.49, 60.9] | 0.079 |
|  | LV | -1.57 [-6.01, 2.86] | 0.477 | -0.696 [-5.57, 4.18] | 0.774 |
| Legend: CI = Confidence Interval, CGMV = Cortical Grey Matter Volume, DGMV = Deep Grey Matter Volume, GMV = Grey Matter Volume,  LC = Lesion Count, LV = Lesion Volume, OR = Odds Ratio, TIV = Total Intracranial Volume, WMV = White Matter Volume | | | | | |

| **SUPPLEMENTARY TABLE 9**  **Variance Inflation Factors for significant linear regression models (GMV, CGMV)** | | |
| --- | --- | --- |
| **Z-score cutoff** | **5-year MRI measure predicted** | **VIF [95% CI]** |
| **Z≤-1.50** | GMV | TIV: 1.24 [1.05, 2.15]  Age: 1.24 [1.05, 2.15]  Sex: 1.28 [1.07, 2.15]  Cognitive outcome: 1.20 [1.04, 2.18] |
|  | CGMV | TIV: 1.24 [1.05, 2.15]  Age: 1.24 [1.05, 2.15]  Sex: 1.28 [1.07, 2.15]  Cognitive outcome: 1.20 [1.04, 2.18] |
| **Z≤-1.00** | GMV | TIV: 1.24 [1.05, 2.15]  Age: 1.24 [1.05, 2.15]  Sex: 1.28 [1.07, 2.15]  Cognitive outcome: 1.20 [1.04, 2.18] |
|  | CGMV | TIV: 1.26 [1.06, 2.15]  Age: 1.20 [1.03, 2.19]  Sex: 1.28 [1.07, 2.15]  Cognitive outcome: 1.20 [1.03, 2.19] |
| Legend: CI = Confidence Interval, CGMV = Cortical Grey Matter Volume, GMV = Grey Matter Volume, TIV = Total Intracranial Volume, VIF = Variance Inflation Factor | | |
